# Stress Effects on the Mechanisms Regulating Appetite in Teleost Fish

**DOI:** 10.3389/fendo.2018.00631

**Published:** 2018-10-23

**Authors:** Marta Conde-Sieira, Mauro Chivite, Jesús M. Míguez, José L. Soengas

**Affiliations:** Laboratorio de Fisioloxía Animal, Departamento de Bioloxía Funcional e Ciencias da Saúde, Facultade de Bioloxía and Centro de Investigación Mariña, Universidade de Vigo, Vigo, Spain

**Keywords:** fish, stress, HPI axis, HSC axis, appetite suppression, food intake regulation, hypothalamic integration

## Abstract

The homeostatic regulation of food intake relies on a complex network involving peripheral and central signals that are integrated in the hypothalamus which in turn responds with the release of orexigenic or anorexigenic neuropeptides that eventually promote or inhibit appetite. Under stress conditions, the mechanisms that control food intake in fish are deregulated and the appetite signals in the brain do not operate as in control conditions resulting in changes in the expression of the appetite-related neuropeptides and usually a decreased food intake. The effect of stress on the mechanisms that regulate food intake in fish seems to be mediated in part by the corticotropin-releasing factor (CRF), an anorexigenic neuropeptide involved in the activation of the HPI axis during the physiological stress response. Furthermore, the melanocortin system is also involved in the connection between the HPI axis and the central control of appetite. The dopaminergic and serotonergic systems are activated during the stress response and they have also been related to the control of food intake. In addition, the central and peripheral mechanisms that mediate nutrient sensing capacity and hence implicated in the metabolic control of appetite are inhibited in fish under stress conditions. Finally, stress also affects peripheral endocrine signals such as leptin. In the present minireview, we summarize the knowledge achieved in recent years regarding the interaction of stress with the different mechanisms that regulate food intake in fish.

## Introduction

**Figure 1:**
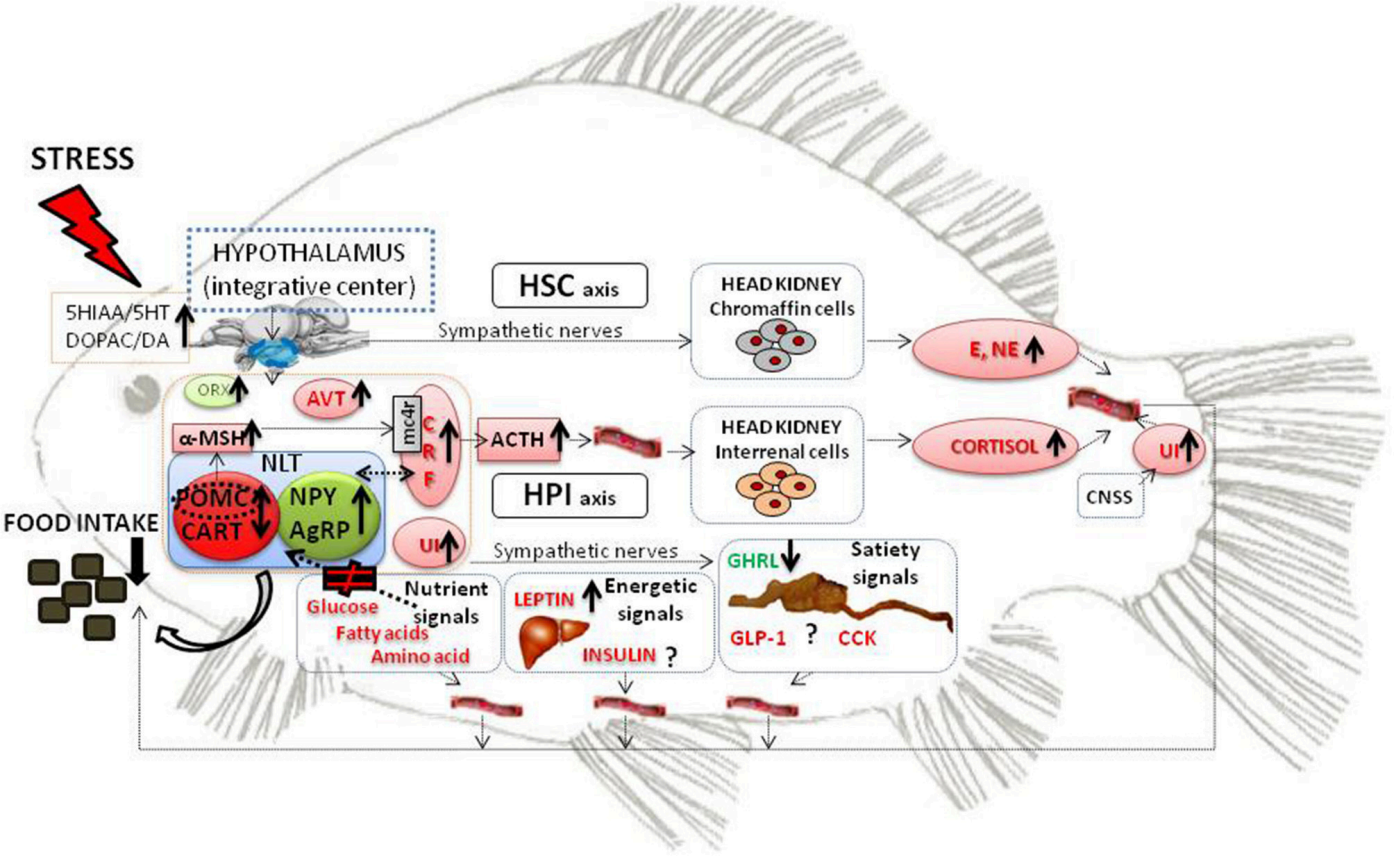
Schematic model representing the main components of the HPI and HSC axis that are activated during the physiological stress response and could be involved in the reduced food intake response under stress conditions. The main factors involved in the homeostatic control of food intake in teleost fish are also represented. Black thick arrows indicate enhanced or decreased levels under stress conditions. Under stress conditions, there is an increase of the dopaminergic and serotonergic activity in the fish brain. HPI and HSC axis are activated leading to the increase of several components with known anorexigenic properties (in red). On the other hand, orexigenic components (in green) are increased (neuropeptides NPY and AgRP) or decreased (ghrelin) under stress conditions. Furhermore, nutrient signals are not properly sensed by the hypothalamus. All this information is integrated in the hypothalamus resulting in decreased food intake. ACTH, adrenocortitropic hormone; AgRP, Agouti-related peptide; AVT, arginine vasotocin; CART, cocaine- and amphetamine-related transcript; CCK, cholecystokinin;CNSS, caudal neurosecretory system; CRF, corticotrophin releasing factor; DA, dopamine; DOPAC, 3,4-dihydroxyphenylacetic Acid; E, epinephrine; GHRL, ghrelin; GLP1 glucagon like peptide 1; HPI, hypothalamus- pituitary-interrenal axis; HSC, hypothalamus-sympathetic system-chromaffin tissue; NE, norepinephrine; NLT, nucleus lateral tuberal; NPY, neuropeptide Y; ORX, orexin; POMC, pro-opio melanocortin; UI, Urotensin I; 5HIAA, 5-hydroxyindole-3-acetic acid; 5HT, serotonin.

In the last years, a great effort has been made in order to describe the mechanisms that regulate food intake in fish (1–3). The inhibition of food intake is a generic response in fish submitted to acute or chronic stress conditions recovering the appetite when the stressor disappears (4–7). In the complex network that regulates appetite, the hypothalamus acts as a central integrator of the endogenous peripheral and central afferent signals informing about the energetic and nutritional status of the animal combined with the incoming external information (1). In the present mini-review, we aimed to describe the existing knowledge about different mechanisms through which stress interacts with food intake control in fish (Figure 1).

## Stress axis activation and its implication on appetite regulation

Stress in fish is an adaptive behavior whose final goal is to reach internal homeostasis after an external disturbance. The response to stress in teleost fish starts with the recognition of an external stimulus (harmful or hazardous) promoting the activation of several neuroendocrine pathways affecting fish physiology (8). Two main pathways have been reported in teleost fish that are activated by a stress stimulus, on one side the hypothalamus-sympathetic system-chromaffin tissue (HSC) involved in the immediate release of catecholamines (CA), epinephrine (E), and norepinephrine (NE) to blood stream and, on the other side, the hypothalamus- pituitary-interrenal axis (HPI). Thus, when fish are submitted to stress, the circulatory levels of CA increase quickly and recover the normal values in a short period of time (minutes) (5, 9, 10).

The control of cortisol synthesis under stress is directly caused by the activation of HPI axis (5). After recognition of a stress stimulus by central nervous system (CNS), corticotropin releasing factor (CRF) is released from hypothalamus stimulating the synthesis and release of adrenocortitropic hormone (ACTH) in the corticotrophs cells of the anterior pituirary gland. Once in the blood stream, ACTH reaches the steroidogenic cells in the interrenal tissue inducing the activation of the melanocortin 2 receptor and therefore stimulating the signaling cascade of synthesis and release of glucocorticoids (11–14). Other neuropeptides that play an important role in the activation of the HPI axis are thyrotropin-releasing hormone (TRH) and arginine vasotocin (AVT), produced and released in hypothalamus, and urotensin I (UI) which is produced in the brain and in terminal segments of the caudal neurosecretory system. TRH, AVT, and UI stimulate the production of cortisol in the interrenal cells directly (UI) or indirectly through promotion of ACTH (UI and AVT) and alpha-melanocyte-stimulating hormone (α-MSH; TRH) (15–19).

In order to understand the anorexigenic effect of the activation of HPI axis, some studies were carried out in fish, suggesting that CRF, UI, AVT, proopiomelanocortin (POMC) via α-MSH, and cortisol play an inhibitory effect on food intake (11, 20, 21, 22, 23, 24). The anorectic effect of stress seems to be mainly mediated by CRF, which is expressed in the telencephalic area, preoptic area, and tuberal hypothalamus (25, 26), matching up with the areas involved in the regulation of food intake. Moreover, several stress stimulus such as hypoxia, handling or isolation, which have a clear anorectic effect, increase the mRNA expression of CRF in those areas (11). The activation of CRF neurons seems to be mediated by α-MSH (among others) through melanocortin 4 receptor (MC4R). In fact, CRF neurons are innervated by melanocortin terminals (27). Thus, once POMC hypothalamic cells are excited, α-MSH is released from the neuronal terminal to the cleft and binds to MC4R receptor in the preoptic area, where is highly expressed, in line with CRF production. Particularly, stress conditions seems to elevate the levels of α-MSH (28), which is directly related to food intake inhibition (29, 30). This is suggesting that the anorexigenic effect of α-MSH involves activation of CRF neurons (30, 31).

In addition, pharmacological experiments demonstrate that CRF and its neuropeptide counterpart UI evoke an anorexigenic effect in teleost fish. UI and CRF are generally recognized as key regulators of the anorexigenic stress response in vertebrates (11) where central injections of UI elicit an anorexigenic effect even more potent than the effect promoted by CRF treatment (22). Furthermore, the effect of this treatment is blocked by the administration of an antagonist of CRF and UI receptor suggesting that these neuropeptides are directly related to the food intake inhibition (32, 33). In addition to this, CRF and UI regulate the presence of ACTH, which works downstream of the melanocortin system (27) and is the main promoter of cortisol release (16, 34, 35). It has been reported that chronic and acute cortisol treatments exert a potent anorexigenic effect in fish (36, 37). Furthermore, cortisol seems to be also involved in maintaining the anorectic response in teleost, with specific glucocorticoid receptors acting as mediators (38). In contrast, in some fish species as goldfish, low levels of cortisol administration have a stimulatory effect on food intake (39) suggesting a different effect of cortisol depending on the species and/or stress severity.

## Monoaminergic systems under stress and their effects on food intake

When fish are submitted to a stress stimulus, several neuronal networks are altered. For instance, serotonergic and dopaminergic systems are fast activated as a consequence of the stress (9). Experiments carried out in rainbow trout demonstrated that the monoaminergic activity in the brain is altered under different kind of stress. For instance, episodes of acute stress stimulated the monoaminergic activity in the forebrain (9, 14). Additionally, some studies evidenced the anorexigenic effect of serotonin and dopamine by using agonist/antagonist of their respective receptors or by stimulation of the monoaminergic activity (4, 40–43).

Moreover, treatments that increase the serotonergic activity also increase the expression of POMC and decrease the expression of neuropeptide Y (NPY) and agouti-related peptide (AgRP) (32, 40, 44). Particularly, the activation of hypothalamic serotonin 5HT_2c_-like receptors (theoretically located in POMC neurons) has an inhibitory effect on food intake in rainbow trout (22, 40, 45).

On the other side, central administration of dopamine induced a decreased gene expression of AgRP whereas there was no effect on POMC and NPY gene expression (43). In the same way, oral treatments with L-Dopa (which is the dopamine precursor) induced an increase of NPY expression but no effects were observed for AgRP and POMC expression in sea bass hypothalamus whereas food intake was inhibited by this treatment (4). These results suggest that the inhibitory effect of dopamine on food intake is not mediated by these hypothalamic neuropeptides. However, the presence of dopamine in fish hypothalamus induces an up regulation of CRF expression, suggesting that the effect of the activation of dopaminergic system under stress conditions on food intake regulation could be mediated by hypothalamic CRF neurons (4).

Furthermore, fish that were previously adapted to a fixed feeding schedule and then subjected to a 7 days fasting period showed increased mRNA abundance of TPH (tryptophan hydroxylase) and TH (tyrosine hydroxylase), which are limitant enzymes of 5HT and DA synthesis. This effect might relate to a feeding/searching behavior instead of the effect of both monoamines on food intake regulation (46).

## The effect of stress on nutrient sensing mechanisms controlling food intake

Nutrient availability can be detected in specialized cells of the organism that possess mechanisms to detect changes in nutrient levels (47). In the last years, nutrient sensing mechanisms have been described in central and peripheral areas of fish, particularly rainbow trout, responding to changes in levels of glucose (48, 49), fatty acids (50), and amino acids (51). The activation of these systems in central areas, especially hypothalamus, relates to the control of food intake through changes in the gene expression of appetite-related neuropeptides (49–53). These nutrient sensing mechanisms are in some way inhibited under stress conditions in fish. Thus, rainbow trout submitted to chronic stress by high stocking density do not respond properly to different glycemic conditions in terms of food intake levels and in the activities/values of parameters involved in glucosensing activity (49, 54). In response to stressors, hypothalamic glucosensing is muted and the regulation of appetite becomes independent of circulating glucose levels. In addition, the decreased gene expression of orexigenic peptides such as NPY and the increased gene expression of anorexigenic factors such as cocaine and amphetamine-related transcript (CART) and POMC observed in hyperglycaemic control fish is not observed in hyperglycaemic fish submitted to crowding stress (52). Furthermore, the expression of these appetite-related neuropeptides in normoglycemic stressed fish are not correlated with a decreased appetite (which would correlate with an increased anorexigenic/decreased orexigenic potential). Altogether, these findings would suggest that the effect of stress on the inhibition of food intake is more related to the loss of the central glucosensing capacity than to a direct regulation of the expression of appetite-related neuropeptides (49, 52). However, the expression of CRF increased in stressed fish irrespective of the glycemic condition (52). In addition, the increase in CRF induces changes in the main parameters related to the glucosensing capacity in rainbow trout hypothalamus. These results suggest that CRF could be involved in the inhibition of the hypothalamic control of food intake by glucosensing mechanisms under stress conditions (55). Stress may also relate to fatty acid sensing mechanisms regulating appetite in fish (47). It has been reported in rainbow trout that the counter-regulatory response to a induced decrease of circulating fatty acid levels is associated with the activation of HPI axis (56, 57). As for the possible effect of stress in the control of food intake exerted by amino acid sensing mechanisms there are no available studies in fish yet.

Moreover, in the last recent years, the regulation of food intake by nutrient sensing mechanisms was reported in fish hypothalamus to be mediated by transcription factors like phosphorylated cAMP response-element binding protein (CREB), forkhead box01 (Fox01), and/or brain homeobox transcription factor (BSX), which would induce variations in the anorexigenic/orexigenic potential in accordance with the presence of nutrients (3). Since stress is known to modulate the expression of the main neuropeptides involved in food intake control in fish, an effect of stress on these transcription factors in fish may not be discarded and clearly deserve to be assessed.

## Interaction of stress with endocrine appetite-related signals

In mammals, insulin and leptin are considered adiposity signals. These peripheral hormones are released to the bloodstream in a way proportional to the white adipose tissue abundance, thus informing the brain about the long-term energetic status of the organism and inducing anorexia (58). In fish, despite this “lipostatic theory” is not so evident since these hormones are more related to glucose homeostasis (59), they have an anorectic effect (60, 61).

Under stress conditions, energy reserves are mobilized to cope with increased metabolic demand, and, therefore, energetic signals related to appetite may be affected. Few studies are available in fish addressing the effect of stress on these energy signals.

Although the role of insulin in food intake regulation in fish is not entirely clear (2), evidence exists regarding an anorexigenic effect of this hormone. Thus, intraperitoneal injection of insulin in rainbow trout decreased food intake whereas isletectomy in goby induced hyperphagia (2). In mammals, it is known that increased glucocorticoid levels results in elevated insulin levels, which leads to Cushing's syndrome associated with type-2 diabetes (62–64). However, there are no available studies in fish regarding the interaction between stress and insulin.

As for leptin, in spite of the low conservation of its gene sequence among animal groups, a similar role of this hormone as energy modulator through mobilization of energy stores during the stress response has been reported in fish as in mammals (60, 65). It has been suggested a synergistic regulation of cortisol and leptin release, with cortisol inducing the leptin release, which in turn elicits the mobilization of energy reserves by stimulating glycogenolysis and gluconeogenesis (60). In fish, elevated cortisol levels induced an increase in hepatic mRNA levels of leptin (37). In turn, ACTH-induced cortisol and ACTH released by CRF induction decrease in the presence of leptin (21, 37, 60, 66). Under hypoxia, mRNA levels of leptin and its receptors in common carp liver increase while food intake decrease, suggesting that leptin is involved in food intake regulation and in the endocrine effects of the stress response (21).

Some studies reported that stress decreases the secretion and availability of the hormones related to the growth hormone (GH)-insulin growth factor-1 (IGF-1) system, the main endocrine growth regulator with implications on food intake regulation (67–69). Thus, the decrease in plasma GH levels has been related to the initial increase of cortisol levels under stress conditions (68). On the other hand, IGF-I levels decreased in plasma of Atlantic salmon exposed to stressful elevated temperatures (69).

Other endocrine signals related to regulation of food intake in teleost fish are also affected by the presence of a stressor. Thus, Pavlidis et al. (70) reported increased levels of orexin (an orexigenic hormone) in high-grade long-term stressed fish, but this hormone seems related to promotion of wakefulness and activity in the presence of stress (70, 71).

In general, ghrelin has been reported to be orexigenic in fish as is in mammals (72–74) by stimulating the expression of NPY and orexin (73, 74). However, the effect of ghrelin on food intake regulation in fish is controversial and depends on fish species, size or via of administration. Thus, some studies reported that ghrelin exerted an anorexigenic effect in fish probably through interaction with CRF neurons (75). In mammals, central ghrelin may play a role in the regulation of food intake during stress (76). In fish, the presence of elevated cortisol levels resulted in a decrease in ghrelin levels in plasma of rainbow trout (77) and tilapia (78). However, in the presence of stress, increased mRNA abundance of ghrelin and its receptors were observed in brain areas of tilapia (79). Ghrelin has been suggested to mediate the decrease in food intake levels in fish under prolonged stress conditions. Moreover, there is not a relationship between ghrelin levels and food intake when fish are submitted to a brief stress, suggesting that other central mechanisms may regulate appetite under short-term stress conditions (79). Accordingly, Cortés et al. (80) reported increased levels of ghrelin mRNA in zebrafish brain after handling stress, suggesting that ghrelin is not mediating the anorexigenic effects of stress or that a rapid counterregulation may occur in response to this anorexigenic effect since increased levels of mRNA of NPY and AgRP were found in hypothalamus in parallel (80). Finally, other appetite-related hormones like cholecystokinin, peptide YY and glucagon-like peptide 1 have anorexigenic properties in fish (1–3) but their interaction with stress response has not been studied yet.

## Effect of stress on the hypothalamic integration of the appetite-related signals

The appetite related signals arriving to the brain are integrated by the hypothalamus which responds in consequence by delivering orexigenic or anorexigenic neuropeptides that eventually induce a decrease or increase in food intake (3). As in mammals, the hypothalamus of fish appears to contain two neuronal populations, AgRP/NPY neurons whose activation resulted in increased food intake (orexigenic potential) and POMC/CART neurons whose activation resulted in decreased appetite (anorexigenic potential). There are several studies in fish addressing the response in hypothalamus of these neuropeptides under stress conditions. Thus, in the presence of stress, increased mRNA levels of hypothalamic NPY were reported in zebrafish submitted to handling stress (80) and rainbow trout under high stocking density (38, 52) or no changes were found in tilapia under both crowding and handling stress (79). In the same way, increased mRNA levels of NPY were found in goldfish after cortisol administration (39), in accordance with that reported in mammals where glucocorticoid treatment increase NPY levels (58). Furthermore, in other brain areas, NPY is also altered under stress conditions. Thus, 24 h treatment with cortisol reduced NPY mRNA abundance in telencephalon but not in hypothalamus of tilapia (78) whereas increased NPY occurred in preoptic area and forebrain in socially subordinate rainbow trout (81). In the case of AgRP, its gene expression increased 15 min after acute handling stress in zebrafish (80). However, these results regarding NPY/AgRP abundance are in contrast with the decreased food intake during stress suggesting that other neuronal population should be mediating the anorectic effect of stress (80). This might relate to the connection of NPY neurons with CRF neurons (39).

Regarding the anorexigenic peptides, CART and POMC mRNA levels are elevated under acute stress conditions in zebrafish (80), but no changes or decreased levels were found in rainbow trout after chronic crowding stress (38, 52). Other studies also found decreased POMC levels in sole under high stocking density (82, 83). These results suggest a complex response depending on the species and the type of stress. Furthermore, the interaction of POMC with stress may be more complex because this peptide is the precursor of other peptides belonging to the melanocortin system that is involved in the activation of the HPI axis as commented previously.

## Conclusions and perspectives

Evidence obtained so far in fish suggest the existence of a complex network through which different components of the stress axis may interact with the mechanisms involved in the regulation of food intake. Despite achievements of recent years, the knowledge regarding these mechanisms is very limited, and basically restricted to the direct actions of cortisol and CRF on neuropeptide expression, the inhibition of glucosensing mechanisms, increased forebrain monoamines, and the interaction with several endocrine systems involved in food intake regulation such as leptin or ghrelin. However, further research is required to address the involvement of the components of the HPI and HSC axis at all levels of food intake control including information arriving from the gastrointestinal tract (impact on chemoreceptors, mechanoreceptors and the flow of information, through nervous system or synthesis and release of gastrointestinal hormones), endocrine systems, and nutrient sensors. Further input is also required to assess the way in which the integration of such information in specific neurons of hypothalamus is altered by different components of the stress axis. Besides elucidation of direct mechanisms, the impact of other factors must be also addressed. These may include the early life stress-effects since it is known in mammals that perinatal stress affects mechanisms regulating food intake at the long term (84). The impact of diet composition in the interaction between food intake control and stress response is also a topic that deserves further research. Finally, almost all studies carried out in fish regarding this subject relate to homeostatic regulation of food intake (3). However, stress might affect not only the mechanisms related to homeostatic control of appetite but also the mechanisms related to hedonic regulation of food intake as in mammals where stress induces increased food intake in animals fed with lipid-enriched meals (85).

## Author contributions

All authors listed have made a substantial, direct and intellectual contribution to the work, and approved it for publication.

### Conflict of interest statement

The authors declare that the research was conducted in the absence of any commercial or financial relationships that could be construed as a potential conflict of interest.
